# Land abandonment and changes in snow cover period accelerate range expansions of sika deer

**DOI:** 10.1002/ece3.2514

**Published:** 2016-10-05

**Authors:** Haruka Ohashi, Yuji Kominami, Motoki Higa, Dai Koide, Katsuhiro Nakao, Ikutaro Tsuyama, Tetsuya Matsui, Nobuyuki Tanaka

**Affiliations:** ^1^ Department of Plant Ecology Forestry and Forest Products Research Institute 1 Matsunosato Tsukuba Ibaraki 305‐8687 Japan; ^2^ Kansai Research Center Forestry and Forest Products Research Institute 68 Nagaikyutaro, Momoyama‐cho Fushimi Kyoto Kyoto 612‐0855 Japan; ^3^ Faculty of Science Kochi University 2‐5‐1 Akebono‐cho Kochi Kochi 780‐8520 Japan; ^4^ Hokkaido Research Center Forestry and Forest Products Research Institute 7 Hitsujigaoka, Toyohira Sapporo Hokkaido 062‐8516 Japan; ^5^ Center for International Partnerships and Research on Climate Change Forestry and Forest Products Research Institute 1 Matsunosato Tsukuba Ibaraki 305‐8687 Japan; ^6^ Center for Global Environmental Research National Institute for Environmental Studies 16‐2 Onogawa Tsukuba Ibaraki 305‐8687 Japan; ^7^ Department of International Agricultural Development Tokyo University of Agriculture 1‐1‐1 Sakuragaoka Setagaya Tokyo 156‐8502 Japan

**Keywords:** *Cervus nippon* Temminck, habitat suitability, human depopulation, neighborhood occupancy, persistence and colonization probability, spatiotemporal change, species distribution modeling

## Abstract

Ongoing climate change and land‐use change have the potential to substantially alter the distribution of large herbivores. This may result in drastic changes in ecosystems by changing plant–herbivore interactions. Here, we developed a model explaining sika deer persistence and colonization between 25 years in terms of neighborhood occupancy and habitat suitability. We used climatic, land‐use, and topographic variables to calculate the habitat suitability and evaluated the contributions of the variables to past range changes of sika deer. We used this model to predict the changes in the range of sika deer over the next 100 years under four scenario groups with the combination of land‐use change and climate change. Our results showed that both climate change and land‐use change had affected the range of sika deer in the past 25 years. Habitat suitability increased in northern or mountainous regions, which account for 71.6% of Japan, in line with a decrease in the snow cover period. Habitat suitability decreased in suburban areas, which account for 28.4% of Japan, corresponding to land‐use changes related to urbanization. In the next 100 years, the decrease in snow cover period and the increase in land abandonment were predicted to accelerate the range expansion of sika deer. Comparison of these two driving factors revealed that climate change will contribute more to range expansion, particularly from the 2070s onward. In scenarios that assumed the influence of both climate change and land‐use change, the total sika deer range increased by between +4.6% and +11.9% from the baseline scenario. Climate change and land‐use change will require additional efforts for future management of sika deer, particularly in the long term.

## 1 Introduction

Large herbivores are major drivers of species diversity and the structure and function of terrestrial ecosystems worldwide (Côte, Rooney, Tremblay, Dussault, & Waller, 2004; Foster, Barton, & Lindenmayer, 2014; Wright et al., 2012). A long history of strong anthropogenic effects on land surfaces has resulted in destructions and fragmentation of the habitats of large herbivores, and many species have now been driven to the brink of extinction (IUCN, 2015). On the other hand, populations of some species have recently increased and caused drastic changes in ecosystems at a national scale (Newson, Johnston, Renwick, Baillie, & Fuller, 2012; Ohashi et al., 2014). These facts indicate that either extinction or overabundance of large herbivores can change the state of ecosystems (Daskin, Stalmans, & Pringle, 2016; Royo, Stout, deCalesta, & Pierson, 2010; Tanentzap, Kirby, & Goldberg, 2012).

Climate change affects the distributions, population dynamics, and body condition of many species (Drever et al., 2012; Kausrud et al., 2008), including large herbivores (Büntgen et al., 2014). Some of the effects can emerge from the direct effects, such as thermoregulation or water limitation, and the indirect effects, such as access to forage or vegetation productivity and quality (Mysterud & Sæther, 2011). In the high‐latitudinal regions, large herbivores often enter negative energy balance in winter because of the deep snow, cold temperatures, and limited forage intake. Ongoing climate change will lead to warmer and shorter winters with less snow in these regions (IPCC, 2013). This would affect the survival and reproduction rates of herbivores by decreasing the energy loss during winter and increasing the length of the growing season for vegetation (i.e., food resources).

Not only the climate change, but also the land‐use change affects the distributions of many organisms worldwide (e.g., Acevedo et al., 2011; Di Marco & Santini, 2015; Kuemmerle, Hickler, Olofsson, Schurgers, & Radeloff, 2012; Levinsky, Skov, Svenning, & Rahbek, 2007; Rondinini & Visconti, 2015). Many developed countries such as Japan are now facing the long‐term human population declines (United Nations, Department of Economic and Social Affairs, Population Division 2015). This human depopulation causes land abandonment, particularly in rural areas. These abandoned fields provide additional habitats for wildlife (Bowen, McAlpine, House, & Smith, 2007; Moreira & Russo, 2007) and are now among the major causes of expansion of the distributions of large herbivores, particularly in developed countries.

The combined effects of climate and land‐use change may act in synergy, or partially counterbalance the negative effects of other drivers of species distribution (Dawe, Bayne, & Boutin, 2014; García‐Valdés, Svenning, Zavala, Purves, & Araújo, 2015). The change in herbivore distribution would eventually result in drastic changes in ecosystem function by altering plant–herbivore interactions (Edenius, Ericsson, Kempe, Bergstrom, & Danell, 2011; Väisänen et al., 2014). The consequences of these complex interactions may not be straightforward and depend on the species and communities. Although there is growing recognition that the effect of altered species interactions sometimes exceeds the direct effect of change in environmental drivers (e.g., Ockendon et al., 2014), this perspective has until recently been overlooked.

**Figure 1:**
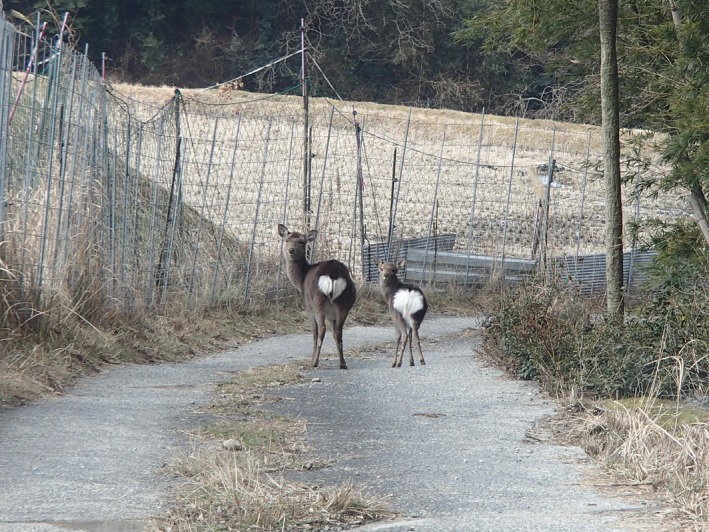
An adult female sika deer (*Cervus nippon* Temminck) and her cub

Japan is one of the world's snowiest regions (Yano, 1993), particularly in the mountainous region along northwest coast. Because Japan's mountainous areas are located at relatively low latitudes (35‐45°N) in a warm temperate zone, even slight climate changes are likely to affect snow conditions (Kawase et al., 2013; Yamaguchi, Abe, Nakai, & Sato, 2011). These changes may cause significant changes in snow‐dominated ecosystem in Japan (Kudo, Amagai, Hoshino, & Kaneko, 2011; Tsuyama et al., 2011, 2012). Moreover, snow is considered important for survival of the sika deer (*Cervus nippon* Temminck, Figure 1), a large herbivore that is causing serious negative impacts on vegetation and ecosystems throughout Japan (Ohashi et al., 2014; Takatsuki, 2009a). Until the 1970s, the distribution of the deer was biased toward the less‐snowy areas with a maximum snow depth of less than about 50 cm (Takatsuki, 1992a). In the 1990s, expansions of the wintering ranges of the deer into snowy areas were reported, and this is suspected to have been facilitated by climate change (Li, Maruyama, Koganezawa, & Kanzaki, 1996).

On the other hand, several reports have emphasized the importance of the effect of land‐use change on the recent range expansion of sika deer (e.g., Agetsuma, 2007; Miyashita et al., 2008). Sika deer prefers to live in landscapes with mixed grassland and forest. Logging increases the availability of forage for sika deer because of the improved light conditions, and these changes last for several decades after logging until the forest regrowth (Takatsuki, 1992b). In addition, a shift in Japan's industrial structure since the 1960s, together with the energy revolution, has caused depopulation of rural areas, and the land abandoned in these areas may have provided forage and shelter for sika deer. The country is now facing a rapid decline in human population, which is leading to the further abandonment of land in rural areas, creating an opportunity for sika deer's range expansion.

Climate change, together with the abandonment of land in rural areas, will likely add to the population growth and range expansions of sika deer, and accelerate the changes in ecosystem function. To minimize the negative effect on ecosystems, it is critical that we develop plans for the strategic management of large herbivores, and these plans are necessary to incorporate the predicted future changes in both climate and land use. Therefore, the assessment of the impacts of climate change and land‐use changes on sika deer populations is essential.

Our first objective here was to statistically evaluate whether the two factors (climate change and land‐use change) were related to the change in range of sika deer in previous decades. If this was the case, it was then important to determine which factor had contributed more to the changes. Our second objective was to use statistical models to predict the future range changes of sika deer under climate change and land‐use change scenarios.

## 2 Material and Methods

### 2.1 Target species

Until the late 19th century, sika deer had been widely distributed across lowlands in Japan. In the 20th century, overexploitation and habitat loss caused by human activity reduced the deer's population and range. Since then, the deer's distribution has been skewed toward mountainous areas with little human activity. After the establishment of legal protection acts in the 1970s, the deer's population recovered, and its geographic range expanded by 170% in 25 years (Ministry of the Environment, 1979, 2004, Figure S1). The animal's negative impacts on forestry, agriculture, and ecosystems have now become problematic (Ohashi et al., 2014; Takatsuki, 2009a). Accordingly, in 2013, the Japanese government decided on a national goal to halve the sika deer population. In addition, in 2014, the Wildlife Protection and Hunting Management Law was revised (Ministry of the Environment, http://www.env.go.jp/nature/choju/index.html), and the government plan was to provide the financial supports to local governments to reduce the sika deer.

Since the 1990s, there were vigorous debates about the likely contributors to the recent range expansion of sika deer. Lack of predators (wild wolves—natural predators of these deer—became extinct in Japan 100 years ago), aging and a decrease in the numbers of hunters, climate change, and land‐use change were the major suspected factors. Several studies have used a species distribution modeling approach to examine the determinant factors of sika deer distribution. In an analysis in Hokkaido, in northern Japan, Kaji, Miyaki, Saitoh, Ono, and Kaneko (2000) pointed out the importance of the effects of snow depth and forage quality. Later, Okumura, Shimizu, and Omasa (2009), in an analysis covering the other three main islands of Japan, revealed the importance of the effects of snow and human land use. However, a recent analysis that covered the whole of Japan found that not climate factors, but anthropogenic factors affected sika deer distribution (Saito, Momose, Inoue, Kurashima, & Matsuda, 2016). Although the last two of these studies considered the effects of the dispersal ability of sika deer by including their distance from their 1970s range, they did not consider the temporal changes in habitat conditions. Accordingly, the importance of temporal changes in habitat condition is still unclear.

### 2.2 Dataset of sika deer distribution

Here, we used the presence–absence data collected across Japan for sika deer in 1978 and 2003 (Ministry of the Environment, 1979, 2004). These surveys were conducted by using questionnaires, interviews, and field survey. The presence or absence of sika deer was recorded in 3′45″ × 2′30″ grid cells (approximately 5 × 5 km^2^, hereafter called “5‐km grid cells”). These data were collected regardless of the season. Respondents to the questionnaires and interviews were forest managers, forest researchers, park rangers, hunters, and members of forest owner cooperatives (Saito et al., 2016). Along with these surveys, an additional survey was conducted after primary data aggregation to prevent an imperfect detection (Ministry of the Environment, 1979, 2004). Therefore, we assumed that the probability of imperfect detection in these surveys was sufficiently small to prevent large biases in the model output. We used data from 15,256 grid cells on the four main islands of Japan (Hokkaido, Honshu, Shikoku, and Kyushu: Figure S1); the total coverage was about 351.8 × 10^3^ km^2^. In 1978, the number of grid cells in which sika deer were recorded as present was 4079, and the number in which they were recorded as absent was 11,177. In 2003, they were recorded as present in 3,800 and absent in 279, of the 4079 grid cells in which they had been recorded as present in 1978. In addition, in 2003, their presence was newly recorded in 3333 additional grid cells in which they had been absent in 2003. In total, in 2003, they were recorded as present in 7133 grid cells and absent in 8123. The number of grid cells in which sika deer were not recorded in both 1978 and 2003 was 7844.

### 2.3 Predictor variables

#### 2.3.1 Climatic variables

We used the snow cover period and the maximum snow depth as candidate climatic variables to explain sika deer distribution. We estimated the snow cover period and the maximum snow depth on 45″ × 30″ grid cells (approximately 1 × 1 km^2^; hereafter referred to as 1‐km grid cells) by using simple degree day method (Kominami, Tanaka, Endo, & Niwano, 2005). Firstly, a daily mean temperature and daily precipitation were calculated in 1‐km grid cells by distance‐weighted mean of three nearest Automated Meteorological Data Acquisition System (AMeDAS) station managed by Japan Meteorological Agency (data were derived from Agriculture, Forestry, and Fisheries Basic Numeric DataBase of AFFRIT, MAFF, Japan). Snow melt coefficient was optimized using satellite data (SPOT Vegetation) (Asaoka & Kominami, 2012, 2013). Daily SWE (snow water equivalent) and then existence of snow cover and snow depth were calculated in 1‐km grid cells; we calculated the mean value in 5‐km grid cells for the analysis, and the mean values between either 1973 and 1977 or 1998 and 2002 were used. Because the Hokkaido subspecies of the deer (*Cervus nippon yesoensis*), which has a large body, seems to have greater tolerance to deep snow (Tokida, Maruyama, Ito, Furubayashi, & Abe, 1980), regional differences between Hokkaido and the other three islands were considered as an interaction term between the climatic variables.

#### 2.3.2 Land‐use variables

We used the proportions of built‐up area, agricultural area, wasteland, and forest area in 1977 and 2002 as land‐use variables. We estimated the proportion of each land‐use type in 1977 and 2002 by temporally interpolating the original data of proportion of each land‐use type in 1976, 1987, 1991, 1997, and 2006 (National Land Numerical Information, Ministry of Land, Infrastructure, Transport and Tourism, http://nlftp.mlit.go.jp/ksj-e/gml/datalist/KsjTmplt-L03-a.html.). We used the inverse distance weight method for interpolation. These data were originally calculated in 1‐km grid cells; we calculated the mean values in 5‐km grid cells for the analysis.

#### 2.3.3 Topographic variables

For other environmental factors, we used the mean slope inclination to consider topographic accessibility, which could affect sika deer distribution by influencing the frequency of human activity (Takii, Izumiyama, Mochizuki, Okumura, & Sato, 2012). These data were originally calculated in 1‐km grid cells; we calculated the mean values in 5‐km grid cells for analysis.

### 2.4 Model construction

**Figure 2:**
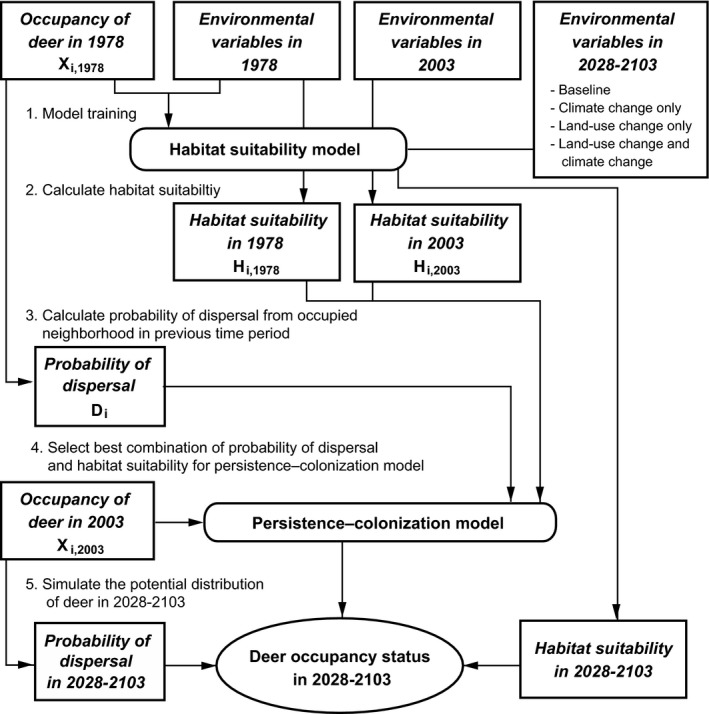
Schematic diagram of modeling procedure used for the persistence–colonization model

We analyzed whether sika deer changed its range according to the estimated changes in suitability surfaces (Figure 2). Sika deer occupancy in 2003 was assumed to be the result of the processes of persistence (a previously occupied cell may still be occupied) and colonization (a previously unoccupied cell may be newly occupied). The occupancy probability of a cell in 2003 was defined as conditional upon the state of occupancy of the cell in 1978, as in the other dynamic occupancy models (Bled, Nichols, & Altwegg, 2013; Yackulic et al., 2012).

If we consider *i *=* *1, 2, …, *N* spatial units (i.e., grid cells), the occupancy *X*_*i,*yr_ (*X*_*i,*yr_ = 1 for presence, 0 for absence) in cell *i* during the survey year (yr), by a Bernoulli distribution with parameter *q*_*i,*yr_ can be denoted as:

$$X_{i,\mathrm{yr}}∼\text{Bernoulli}(q_{i,\mathrm{yr}})$$

The probability of occurrence of sika deer in 2003 was derived from the occupancy status in 1978 and by a dynamic process of colonization and extinction as follows:

$$q_{i,2003}=\varphi_{i}X_{i,1978}+\gamma_{i}\left(1-X_{i,1978}\right)$$

where φ_*i*_ and γ_*i*_ are the persistence and colonization probabilities, respectively, for cell *i* between 1978 and 2003. We defined these probabilities by using two components: (1) the probability of immigration from neighboring cells during one time period (probability of dispersal, *D*_*i*_) and (2) the species’ preference for the cell's environment (habitat suitability, *H*_*i*_) (Lawes, Mealin, & Piper, 2000). Therefore, we defined the persistence (φ_*i*_) and colonization (γ_*i*_) probabilities as:

$$\text{logit}\left(\varphi_{i}\right)=\varphi_{0}+\varphi_{d}D_{i}+\varphi_{h}H_{i}$$

$$\text{logit}\left(\gamma_{i}\right)=\gamma_{0}+\gamma_{d}D_{i}+\gamma_{h}H_{i}$$

where φ_0_ and γ_0_ are intercepts, φ_*d*_ and γ_*d*_ are slopes for the probability of dispersal *D*_*i*_, and φ_*h*_ and γ_*h*_ are slopes for habitat suitability *H*_*i*_.

We assumed that the value of *D*_*i*_ was determined by neighborhood occupancy during the previous time period. A cell that has a large number of occupied neighbors is more likely to stay occupied, or to be colonized, than to become unoccupied. We tested the combinations of five patterns of neighborhood radius (10, 25, 50, 75, and 100 km) and three patterns of assignment of weight to grid cells within the neighborhood (equal weight, weighted by inverse distance, and weighted by square of inverse distance), which gave 15 candidate neighborhood occupancy values. Although there are several other methods to separate the dispersal from habitat effects (Broms, Hooten, Johnson, Altwegg, & Conquest, 2016; Sutherland, Elston, & Lambin, 2012), this method has the advantage of low computational intensity (Dormann et al., 2007) and allows the application of the model to a large dataset that covers a large spatial extent with fine resolution.

To obtain an optimal habitat suitability surface to explain the change in range of sika deer between 1978 and 2003, we used the distribution data for sika deer and environmental data in 1978 to train the multiple candidate models to obtain the habitat suitability surfaces:

$$\text{logit}\left(q_{i,1978}\right)=\alpha +\beta_{1}x_{1,i,1978}+\beta_{2}x_{2,i,1978}+\ldots +\beta_{j}X_{j,i,1978}$$

where α is intercept and β_*j*_ are coefficient parameter of *j*th environmental variable, *x*_*j*._

These candidate models include all possible combinations of climatic variables, land‐use variables, and topographic variables. To include the nonlinear relationship between sika deer occurrence and habitat conditions, we checked the generalized additive model fitting curves and decided to include a second‐order term for the proportion of agricultural land. We also compared the predictive performances of two incompatible climatic variables, namely the snow cover period and the maximum snow depth. From 16 candidate combinations of all variables, we excluded the model with both the snow cover period and the maximum snow depth to avoid the effect of multicollinearity and nominated 12 candidate habitat models for further analysis (Table S1). Because the presence and absence of sika deer may not be at equilibrium as a result of dispersal limitation, we also considered the models with autocovariates. We considered two patterns of neighborhood radius (10 and 25 km) and three patterns of assignment of weight to grid cells within the neighborhood for autocovariates, which resulted in 84 candidate models.

Subsequently, we used the environmental variables in 1978 and 2003 to calculate a habitat suitability surface for 84 habitat suitability models in each survey period, namely habitat suitability surface in 1978 (*H*_1978,*i*_) and 2003 (*H*_2003,*i*_). A grid cell with a relatively high suitability value was likely to stay occupied or to be preferentially colonized by sika deer. We defined habitat suitabilities as the log odds (probability on a logit scale) of predictions from the fitted models:

$$H_{1978,i}=\alpha +\beta_{1}x_{1,i,1978}+\beta_{2}x_{2,i,1978}+\cdots +\beta_{j}X_{j,i,1978}$$

$$H_{2003,i}=\alpha +\beta_{1}x_{1,i,2003}+\beta_{2}x_{2,i,2003}+\cdots +\beta_{j}X_{j,i,1978}$$

Combination of the two survey periods and 84 habitat suitability models resulted in 168 candidate habitat suitability surfaces.

By using the program WinBUGS (Lunn, Thomas, Best, & Spiegelhalter, 2000), we used Bayesian modeling and Markov chain Monte Carlo posterior sampling to estimate the parameters of the dynamic‐range change model to explain the sika deer presence in 2003. We ran three chains, using noninformative priors, for 1,000 iterations after a 100‐iteration burn‐in period. Overall, we compared the Watanabe–Akaike Information Criteria (WAIC) (Watanabe, 2010) of 2,520 competing models and selected the model with the minimum WAIC value, which indicated the best combination of neighborhood occupancy and habitat suitability to explain the range dynamics between 1978 and 2003. If the change in the range of sika deer corresponded to the change in habitat suitability as a result of climate change and land‐use change in 25 years, habitat suitability in 2003 should be selected. If not, habitat suitability in 1978 should be selected. The habitat suitability surface will be selected by explanatory power for sika deer range dynamics between 1978 and 2003, and it is not necessarily always the optimal model to explain the presence or absence of sika deer in 1978, as criticized by Yackulic, Nichols, Reid, and Der (2015).

### 2.5 Future climate change and land‐use change scenario for the future

For future predictions, we used four environmental scenario groups: (1) baseline, (2) land‐use change only, (3) climate change only, and (4) land‐use change and climate change, from 2004 to 2103 (Table S2). Baseline is a scenario of no change in climate and land use.

Land‐use change only scenario group was made from nine land‐use change scenarios (Hanasaki, Takahashi, & Hijioka, 2012) based on different patterns of future human population projection. The human population projections were made by combining three patterns of birth rate and death rate—high birth rate and low death rate, medium birth rate and medium death rate, and low birth rate and high death rate—and three patterns of spatial distribution pattern, namely centralization, baseline, and decentralization (Ariga & Matsuhashi, 2012). In this scenario, the relationship between human population density in 2005 (2005 Population Census, Ministry of Internal Affairs and Communications, http://www.stat.go.jp/data/kokusei/2005/index.htm) and the built‐up area in 2006 (National Land Numerical Information, Ministry of Land, Infrastructure, Transport and Tourism, http://nlftp.mlit.go.jp/ksj-e/gml/datalist/KsjTmplt-L03-a.html.) was modeled by using a simple linear regression analysis at a 1‐km grid cell resolution. Then, future land‐use patterns were predicted with the model according to the predicted future human population (Ariga & Matsuhashi, 2012; National Institute of Population and Social Security Research, 2008). If the built‐up area was predicted to increase, the areas of agricultural land, forests, and wasteland would decrease. If the built‐up area was predicted to decrease, “wastelands” and “other land uses” were additionally allocated, assuming that land abandonment occurred (Hanasaki et al., 2012). We calculated the mean value of each land‐use type in 5‐km grid cells, and we used these values for future projection.

Climate change only scenario group was made from four scenarios of the snow cover period and the maximum snow depth, which are calculated from the data of four general circulation models (GCMs) of the World Climate Research Program's Coupled Model Intercomparison Project phase 3 multimodel dataset: CSIRO‐Mk3.0, MRI‐CGCM2.3.2a, GFDL‐CM2.1, and MIROC3.2 (high resolution). The velocity of climate change is slowest in CSIRO‐Mk3.0, medium in MRI‐CGCM 2.3.2a and GFDL‐CM 2.1, and fastest in MIROC 3.2 (high resolution). The A1B scenario (720‐ppm stabilization experiment) from the Special Report on Emissions Scenario (SRES) in 2021–2025, 2046–2050, 2071–2075, and 2096–2100 was used to simulate the future climate, and the Climate of the 20th‐century experiment (20c3 m) in 1980–1999 was used as a baseline for the current climate. Climate simulation datasets from these two experiments for the four GCMs were obtained from the IPCC Data Distribution Centre (http://www.ipcc-data.org/sim/gcm_monthly/SRES_AR4/index.html, last accessed 1 June 2012). Differences in mean monthly temperature and the ratio of monthly precipitation flux between current and future climate were spatially interpolated into 1‐km grid cells. These differences and ratios for the two climate datasets were then overlaid on a snowfall reconstruction model based on the data for 1980–1999, and the mean values of 20‐year snowfall reconstruction with overlaid climate were used to determine the future climate under each of the four GCMs.

Land‐use and climate change scenario group was made from all possible combination of nine scenarios of land‐use change and four scenarios of climate change, which resulted in 36 scenarios.

To predict the potential future changes in the range of sika deer, the model that we developed was used to project deer distributions in 2028, 2053, 2078, and 2103. Because our aim was to clarify the difference between land‐use change and climate change scenarios, we used the median values of each coefficient in our model. First, the probability of sika deer occurrence in 2028 was calculated on the basis of the neighborhood occupancy status in 2003. We then used the probability of occurrence of sika deer in 2028 as a weighting to obtain the probability of the sampling indicating the deer's presence, and we randomly allocated the occupancy status of each grid cell in 2028 as present (*X*_*i*,2028_ = 1) or absent (*X*_*i*,2028_ = 0). We used this allocated occupancy status to generate autologistic covariates and calculated the probability of sika deer occurrence in the next time period; we repeated this process until 2103. We performed the simulation 1,000 times. For each grid cell, we counted the number of trials for which the occupancy status was present and divided the result by 1,000 to obtain the probability of that cell being occupied by sika deer at each time step. Because there is a possibility of failure to detect the presence of sika deer at lower abundance, such as at range boundaries, we defined the potential distributions of sika deer using the 2003 threshold value, with a sensitivity value of 95%, which gives greater importance to avoid false negatives (Newbold, Gilbert, Zalat, El‐Gabbas, & Reader, 2009). We considered those grid cells with a probability of being occupied by sika deer as larger than the threshold value as part of the potential distribution of the deer at each time step and used them to calculate the total area of potential distribution.

We conducted simulations for the combinations of five climate change scenarios and 10 land‐use change scenarios. We defined the combination of baseline climate change scenario and baseline land‐use change scenario as “baseline scenario (*n *=* *1).” We defined the combinations of nine different land‐use change scenarios and a baseline climate change scenario as the “land‐use change only scenario (*n *=* *9)”; the combinations of a baseline land‐use change scenario and four different climate change scenarios as “climate change only scenario (*n *=* *4)”; and the combination of nine different land‐use change scenarios and four different climate change scenarios as “land‐use and climate change scenario (*n *=* *36).” The total areas of the potential distributions of sika deer were compared among the four groups of future land‐use and climate change scenarios. To reflect the uncertainty within each scenario group, we compared the average value of predicted distribution, which ranges between 0 and 1, and the average total area of the potential distribution of sika deer. We used the “mgcv,” “spdep,” “R2WinBUGS,” and “ROCR” packages in R 3.2.0 (R Development Core Team, 2015). ArcMap 10.2 (ESRI Inc.) and QGIS 2.6.1 (http://www.qgis.org/ja/site/) were used in the analytical procedure.

## 3 Result

### 3.1 Drivers of change in the range of sika deer in the last 25 years

In the top five models with the lowest WAIC values, we used land‐use variables, climatic variables, and topographic variables to calculate habitat suitability, excluding any autocovariate (Table 1). Within the climatic variables, the snow cover period was selected in the top five models, but the maximum snow depth was not selected in any of these models. In addition, when comparing the models with same distance and weights, the models that used habitat suitability surface in 2003 had lower WAIC values than those that used habitat suitability surface in 1978 (Table 1), which indicates more high explanatory power to explain sika deer range in 2003. A neighborhood radius of 100 km was selected in two models, 75 km was selected in two models, and 50 km was selected in one model. All of the top five models selected inverse‐squared weighting methods. The area under curve of our final model was 0.933 (Table 1). The threshold value of probability of occurrence for defining the potential distribution of sika deer was 0.1725.

**Table 1:** Formulae of the top five habitat suitability models, as well as years used to calculate the habitat suitability; variables, distances, and weights used to calculate autocovariates, and Watanabe–Akaike Information Criteria (WAIC) and area under the curve (AUC) for these models

Rank	Distance (km)	Weight	Habitat suitability model	Year	WAIC	AUC
1	100	Inverse‐squared	~LU + CL[SCP] +TOPO	2003	0.3288	0.9334
2	75	Inverse‐squared	~LU + CL[SCP] +TOPO	2003	0.3301	0.9329
3	100	Inverse‐squared	~LU + CL[SCP] +TOPO	1978	0.3310	0.9325
4	75	Inverse‐squared	~LU + CL[SCP] +TOPO	1978	0.3321	0.9321
5	50	Inverse‐squared	~LU + CL[SCP] +TOPO	2003	0.3328	0.9317

LU, land‐use variables; CL, climatic variables; TOPO, topographic variables; SCP, snow cover period.

Within the parameters of the range change model, the coefficients for probability of dispersal and habitat suitability were positive in both the persistence model and the colonization model (Table 2). The coefficient for the probability of dispersal was larger in the colonization model than in the persistence model, whereas the coefficient of habitat suitability did not differ markedly between persistence and colonization.

**Table 2:** Parameters used in the range change model. See text for definitions

	Mean	*SD*	2.5%	25%	50%	75%	97.5%	Rhat
φ_0_	–0.04	0.17	–0.36	–0.15	–0.04	0.07	0.31	1.00
φ_*d*_	5.95	0.34	5.26	5.72	5.94	6.17	6.62	1.00
φ_*h*_	0.50	0.06	0.38	0.46	0.50	0.54	0.63	1.00
γ_0_	–1.34	0.05	–1.43	–1.37	–1.34	–1.31	–1.25	1.00
γ_*d*_	8.29	0.20	7.90	8.15	8.28	8.42	8.69	1.00
γ_*h*_	0.64	0.02	0.60	0.63	0.64	0.66	0.68	1.00
Deviance	10018.92	7.06	10,010	10,010	10,020	10,020	10,030	1.00

φ_0_, intercept in persistence model; φ_*d*_, coefficient for the probability of dispersal in persistence model; φ_*h*_, coefficient for the habitat suitability in persistence model; γ_0_, intercept in colonization model; γ_*d*_, coefficient for the probability of dispersal in colonization model; γ_*h*_, coefficient for the habitat suitability in colonization model

In the best habitat suitability model, sika deer showed a negative response to snow cover period; this was less significant (i.e., more weakly negatively correlated) in Honshu, Shikoku, and Kyushu, as indicated by the interaction term (Table 3). Sika deer showed positive responses to slope inclination, forest cover, and wasteland cover and negative responses to built‐up area (Table 3). Unimodal responses to agricultural land were also confirmed.

**Table 3:** Parameter estimates of the best model for calculating the habitat suitability for sika deer. Lower and upper 95% confidence intervals (95% CI) are shown. “Hokkaido” is set as the reference category for the variable “region”

Category	Variables	Coefficient	Lower 95% CI	Upper 95% CI
	Intercept	1.236	–0.479	2.951
CL	SCP	–0.036	–0.040	–0.032
CL	RGN (the other three islands)	–6.511	–7.146	–5.876
CL	SCP × RGN (the other three islands)	0.020	0.016	0.024
LU	FR	4.273	2.566	5.980
LU	WS	3.623	1.818	5.428
LU	AG	5.264	3.451	7.077
LU	AG^2^	–3.061	–4.088	–2.034
LU	BT	–2.894	–5.738	–0.050
TOPO	SL	0.115	0.103	0.127

LU, land‐use variables; CL, climatic variables; TOPO, topographic variables; SCP, snow cover period; SL, slope inclination; WS, the proportion of wastelands; AG, the proportion of agricultural lands; FR, the proportion of forests; BT, the proportion of built‐up areas; RGN, region.

**Figure 3:**
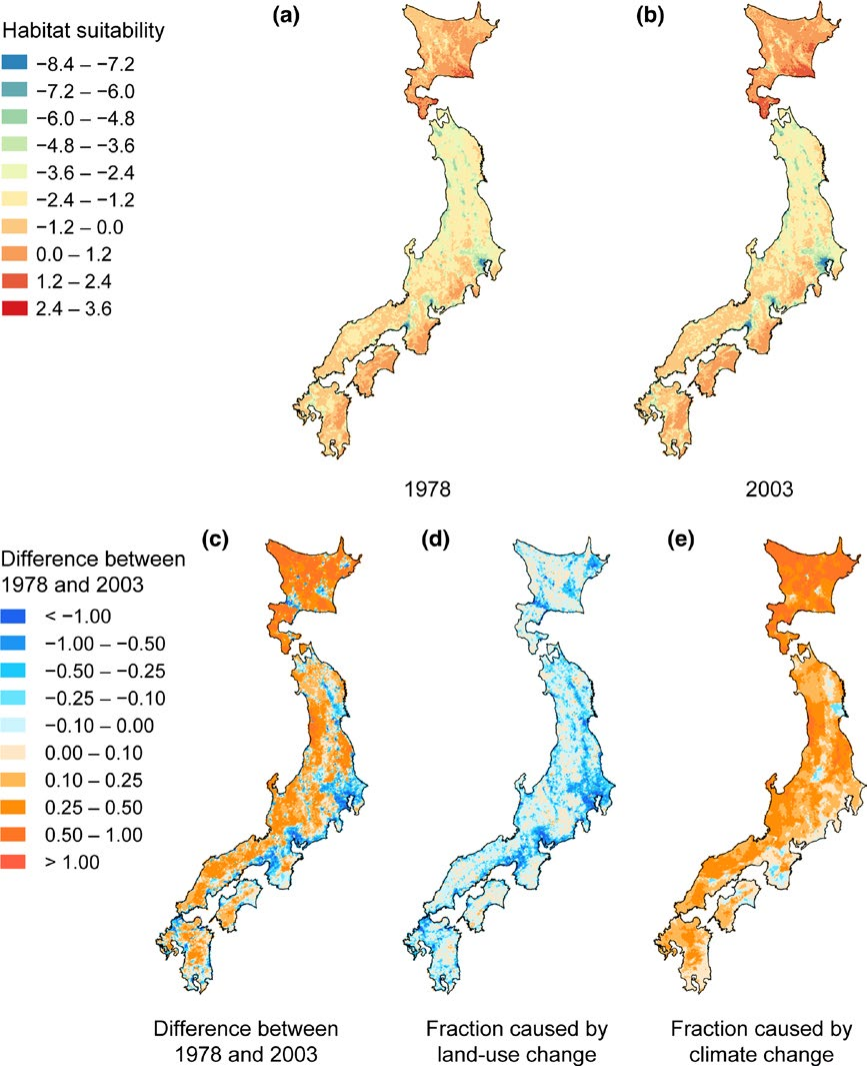
Map of habitat suitability in (a) 1978 and (b) 2003. (c) Difference between 1978 and 2003, (d) the fraction of land‐use change between 1978 and 2003, and (e) the fraction of climate change between 1978 and 2003

From 1978 to 2003, habitat suitability for sika deer increased in 10,925 grid cells, accounting for 71.6% of the total area of Japan, mainly in the northern and mountainous regions (Table 4, Figure 3a–c). In contrast, habitat suitability decreased in 4,331 grid cells, accounting for 28.4% of the total area, mainly in suburban areas (Table 4, Figure 3a–c). Most of the habitat suitability increment was caused by climate change (Figure 3e). Most of the habitat suitability decrease was caused by land‐use change (Figure 3d). Habitat suitability decreased markedly in areas surrounding large cities; this indicated habitat loss was caused by urbanization from 1978 to 2003 (Figure 3d).

**Table 4:** Trend of change in habitat suitability between 1978 and 2003 (the numbers of cells in which it increased or decreased), and the relative contribution of land‐use change and climate change (LU > CL: the absolute value of the changes in habitat suitability caused by land‐use change was larger than that caused by climate change; LU < CL: the absolute value of the change in habitat suitability caused by climate change was larger than that caused by land‐use change). χ^2^‐value = 12,697, df = 1, *p*‐value < .001

	Habitat suitability (no. of grid cells)	
	Increased	Decreased
Absolute value of difference in contribution		
LU > CL	220	4011
LU < CL	10,705	320
Total	10,925	4331

### 3.2 Predicted future range changes of sika deer in the next 100 years

**Figure 4:**
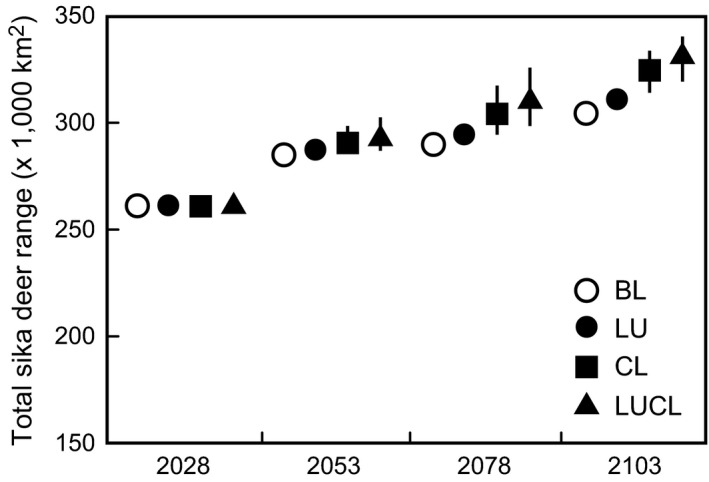
Total sika deer ranges predicted in 2028, 2053, 2078, and 2103. Open circle: baseline scenario (BL); closed circle: scenario of land‐use change only (LU); squares: scenario of climate change only (CL); triangles: scenario of both land‐use and climate change (LUCL). Bars show 95% percentiles

**Figure 5:**
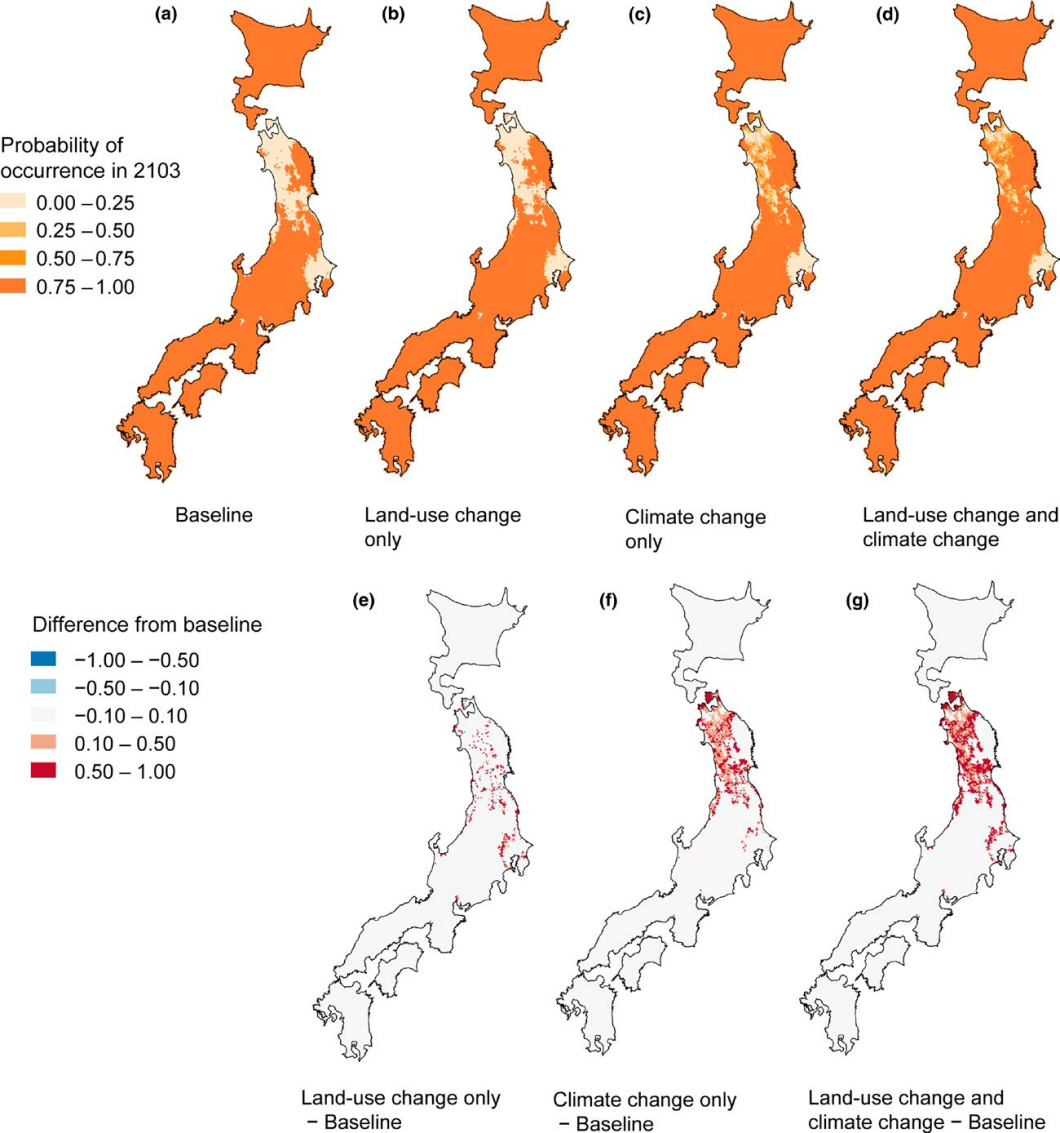
Mean probabilities of occurrence of sika deer in 2013 under four scenario groups: (a) baseline scenario; (b) scenario of land‐use change only; (c) scenario of climate change only; (d) scenario of both land use and climate change. Also (e) difference between baseline scenario and scenario of land‐use change only; (f) difference between baseline scenario and scenario of climate change only; and (g) difference between baseline scenario and scenario of both land‐use change and climate change

The result of our simulation of future changes showed the considerable range expansion of sika deer in the coming 100 years. In 2028, the effects of climate change and land‐use change on deer range would be relatively small (Figure 4). However, the differences among the four groups of future climate change and land‐use change scenarios would become apparent from 2078 onward. In the baseline scenario, sika deer would expand its range by 304.2 × 10^3^ km^2^ in 2103 (Figure 5a). In the “land‐use change only” scenario, the total range would increase by 308.5 × 10^3^ to 312.1 × 10^3^ km^2^, a + 1.4% to +2.6% increase over the baseline scenario (Figure 5b). Compared with the baseline scenario, the deer range would expand around suburban areas (Figure 5e). In the “climate change only” scenario, the total sika deer range would increase by 313.7 × 10^3^ to 334.0 × 10^3 ^km^2^, resulting in a + 3.1% to +9.8% increase over the baseline scenario (Figure 5c). Compared with the baseline scenario, the deer range would expand in northern Honshu (Figure 5f). In the “land‐use and climate change” scenario, the total sika deer range would increase by 318.3 × 10^3^ to 340.4 × 10^3^ km^2^, which result in +4.6% to +11.9% increase over baseline scenario (Figure 5d). Range expansion was predicted to occur both in areas surrounding the suburbs and in northern Honshu (Figure 5g).

## 4 Discussion

### 4.1 Which of these two factors—land‐use change or climate change—has contributed more to the range change of sika deer in recent decades?

We found here that habitat suitability of sika deer was explained by a combination of climatic variables, land‐use variables, and topographic variables (Table 1).

Our results revealed the importance of the snow cover period compared with the maximum snow depth, which had been used in previous studies (Kaji et al., 2000; Okumura et al., 2009; Saito et al., 2016). In snowy regions in Japan, evergreen dwarf bamboo species dominate on the forest floor (Tsuyama et al., 2011, 2012), and snow cover prevents the access to this forage. Therefore, the snow cover period directly affects the amount of forage available in winter (Minamino & Akashi, 2011). This indirect effect of climate might be a more important determinant of the distribution of sika deer than are direct effects of climate such as the energy cost of moving in deep snow (Parker & Robbins, 1984).

The response of sika deer distribution to land‐use variables (Table 3) was consistent with the other previous studies. The positive responses to forest and wasteland and the unimodal response to agricultural land may indicate the preference for forest, grassland, and forest–agriculture ecotones (Saito et al., 2016). Sika deer tend to avoid built‐up areas, owing to the frequent human disturbance (Okumura et al., 2009; Saito et al., 2016). In our study, slope inclination was selected as an important variable. In general, deer avoid using steep slopes to minimize unnecessary energy loss (Ganskopp & Vavra, 1987; Moen, 1976). However, in the mountainous areas of Japan, deer find steep slope safe, with little human disturbances, during the hunting season (Takii et al., 2012). In this regard, our result agrees with those of this earlier study and may reflect the indirect effect of human disturbance, which had been overlooked in previous macroscale studies.

We found here that both climate change and land‐use change between 1978 and 2003 have affected the changes in deer range (Figure 3). Because sika deer have high mobility, they can change their distribution correspondingly to the spatiotemporal change in habitat condition, rather than stick to previous habitat condition. Our result indicated that a decrease in snow cover period in the last few decades has increased the habitat suitability of sika deer throughout most of the Japanese islands—especially in northwestern Japan and in mountainous regions. In the snowy area, sika deer migrate between separate summer and winter ranges to avoid unsuitable climatic condition, while it becomes sedentary in the area with no snow (Takatsuki, 2009b; Yabe & Takatsuki, 2009). Under a favorable climate, there is no need for seasonal migrants to return to their winter ranges, so they tend to stay in their summer ranges. These individuals become the founders of new populations, and this leads to further range expansion (Maruyama, 1981). The short snow cover period in recent years may have contributed to the expansion of populations in habitats that were previously unsuitable because of climatic conditions.

In contrast, changes in land use resulted in a decrease in habitat suitability of sika deer at suburban area and in a slight increase in mountainous area. Decrease in habitat suitability in suburban area was mainly due to the decrease in forest and the increase in built‐up area. Slight increase in habitat suitability in mountainous area was mainly due to the decrease in wasteland and the increase in forest.

Our results also revealed that the response to changes in snow cover period differed between Hokkaido and the other islands. Habitat suitability was greater in Hokkaido than on the other three islands (Table 3). Additionally, we found a more significant effect of the snow cover period in Hokkaido, as indicated by the interaction term (SCP × RGN in Table 3). Both ecological and historical differences between two regions are expected to cause the difference in the sensitivity of populations to changes in the snow cover period. As ecological factor, Hokkaido population has the greater tolerance to deeper snow due to its large body size, compared with the population in other three islands. Release from suppression by a long snow cover period might result in explosive range expansion as a reaction, as we have observed in past decades (Kaji et al., 2000). As historical factor, human land use is more intense in Honshu, Shikoku, and Kyushu compared with Hokkaido, which have started to develop in the late 1800s. Long history of intense human land use may have resulted in local extinction of sika deer, even in the areas of suitable habitat. Additionally, human land‐use footprint may have the potential to increase the persistence of ungulates under severe winter conditions, for example, by increasing the amount of available forage (Dawe et al., 2014). However, process and mechanism to cause the difference between two region, and the relative importance of these two factors, are still unknown. More detailed research is required to clarify the factors responsible for the population differences between Hokkaido and the other islands.

### 4.2 Effects of future land‐use change and climate change on the range of sika deer

We found here that the effects of climate change and land‐use change were minimal in 2028, although we had predicted that the sika deer range would expand (Figures 4 and 5). However, the effects of land‐use change and climate change became more substantial from 2078 onward (Figures 4 and 5). Both land abandonment caused by human depopulation and the decrease in snow cover period caused by climate change had predicted to accelerate the deer's range expansion, and combining the two drivers gave the largest rate of range expansion. In one study in Europe (Rondinini & Visconti, 2015), populations of red deer (*Cervus elaphus* Linnaeus)—a species that is closely related to sika deer and is distributed over the Eurasian continent—were also predicted to increase under the predicted climate change and land‐use change, unlike the case with other large herbivores, such as moose (*Alces alces* Linnaeus) and reindeer (*Rangifer tarandus* Linnaeus), which inhabits in more northern biome. These differences in response to climate change relate to tolerance to heat condition. Previously, the southern limit of sika deer was at northern Vietnam in the tropical environment, which has now become extinct in the wild (McCullough, 2009). Sika deer have relatively high tolerance to high temperature and may receive the positive effect after climate change, at least in Japan.

There are many predictive studies that have reported the risks of species range loss and extinctions as a result of climate change and land‐use change (Rondinini & Visconti, 2015). However, most of these works only consider the direct effect of change in driver and fail to notice the indirect effect of change in biotic interactions, such as the intensification of deer impact on vegetation. For example, Siebold's beech (*Fagus crenata* Blume)—the dominant tree in the cool temperate zone of Japan—is projected by some models to decrease its potential habitats widely according to the future climate change (Koide et al., 2016; Matsui et al., 2004, 2009; Nakao et al., 2013). Additionally, the regeneration of *Fagus crenata* would become suppressed under heavy browsing by sika deer (Takatsuki & Gorai, 1994). Our result indicates that climate change and land‐use change may add further risk for *Fagus crenata* by increasing the area with heavy browsing by deer in the next 100 years. Heavy browsing by deer may eliminate some vulnerable plant species, such as *Fagus crenata*, and enhance the range expansion of other tolerant species. Effect of climate change and land‐use change on the species distribution may be accelerated, or decelerated, depending on their vulnerability to deer browsing. To predict the future vegetation, we need to take account for the synergistic effect of changes in climate, land‐use, and intense impact of deer browsing on vegetation.

Our results revealed that the effects of climate change and land‐use change may strengthen after the 2050s. Although the effects of both drivers are not likely to result in short‐term consequences, if we overlook their effects, we may underestimate the effort required to control deer populations, particularly over the long term. The sika deer range in northern Honshu and in mountainous areas may particularly be strongly influenced by climate change in the next 100 years. Building our capacity for monitoring and population control in these areas may help with early detection of range expansion in the front line and thus with the rapid population control.

## Conflict of Interest

None declared.
