# Supplementary material for: Land abandonment and changes in snow cover period accelerate range expansions of sika deer
Source: Ecol Evol. 2016 Oct 5;6(21):7763–75. doi: 10.1002/ece3.2514 (PMC6093158; doi:10.1002/ece3.2514)

Fig. S1

Map of sika deer distributions in Japan in each observation year (after Ministry of the Environment 1979, 2004).


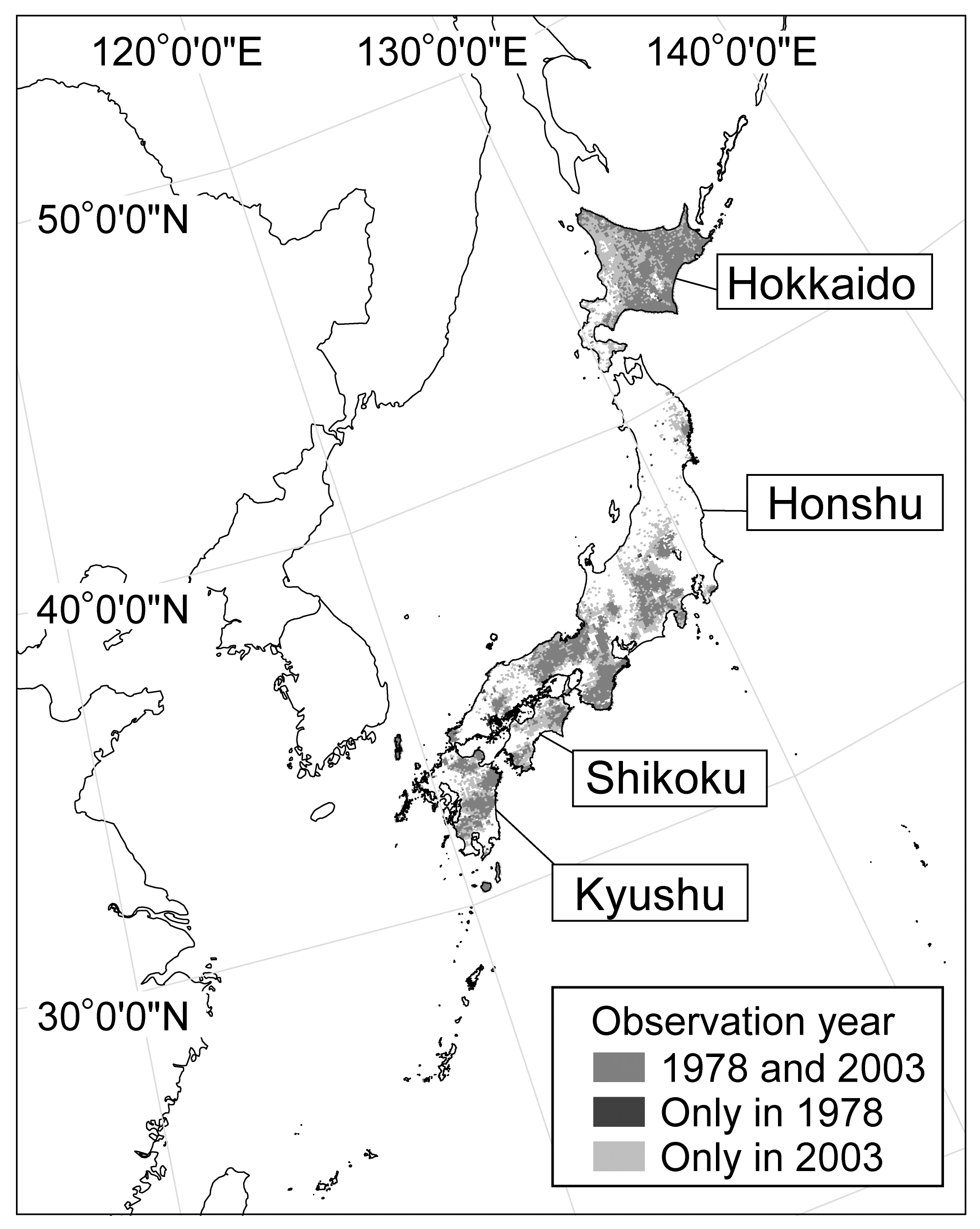

Supplement: Supplementary file 1 [file ECE3-6-7763-s001.doc]
