# Supplementary material for: Land abandonment and changes in snow cover period accelerate range expansions of sika deer
Source: Ecol Evol. 2016 Oct 5;6(21):7763–75. doi: 10.1002/ece3.2514 (PMC6093158; doi:10.1002/ece3.2514)
Supplement: Supplementary file 2 [file ECE3-6-7763-s002.docx]

Table S1. List of 12 candidate habitat models.

|  |  |  | |  |  |  |  |
| --- | --- | --- | --- | --- | --- | --- | --- |
| Model ID | Climatic variables | | |  | Land-use |  | Topographic |
|  | SCP+RGN  +SCP×RGN | | MSD+RGN  +MSD×RGN |  | FR+WS+AG+AG^2^+BT |  | SL |
| 1 |  | |  |  |  |  |  |
| 2 | X | |  |  |  |  |  |
| 3 |  | | X |  |  |  |  |
| 4 |  | |  |  | X |  |  |
| 5 | X | |  |  | X |  |  |
| 6 |  | | X |  | X |  |  |
| 7 |  | |  |  |  |  | X |
| 8 | X | |  |  |  |  | X |
| 9 |  | | X |  |  |  | X |
| 10 |  | |  |  | X |  | X |
| 11 | X | |  |  | X |  | X |
| 12 |  | | X |  | X |  | X |
