# Supplementary material for: Land abandonment and changes in snow cover period accelerate range expansions of sika deer
Source: Ecol Evol. 2016 Oct 5;6(21):7763–75. doi: 10.1002/ece3.2514 (PMC6093158; doi:10.1002/ece3.2514)
Supplement: Supplementary file 3 [file ECE3-6-7763-s003.docx]

Table S2. Correspondence table of scenario groups and combination of climate change scenario and land-use change scenario.

|  |  |  | Climate change scenario | | | | |
| --- | --- | --- | --- | --- | --- | --- | --- |
|  |  |  | Baseline | CSIRO- Mk3.0 | MRI-CGCM2.3.2a | GFDL- CM2.1 | MIROC3.2  (high resolution) |
| Land-use  change scenario | Baseline |  | **Baseline** | **Cllimate change only** | | | |
|  | High birth and low death | - centralization | **Land-use change only** | **Land-use change and climate change** | | | |
|  |  | - baseline |  |  |  |  |  |
|  |  | - decentralization |  |  |  |  |  |
|  | Medium birth and medium death | - centralization |  |  |  |  |  |
|  |  | - baseline |  |  |  |  |  |
|  |  | - decentralization |  |  |  |  |  |
|  | Low birth and high death | - centralization |  |  |  |  |  |
|  |  | - baseline |  |  |  |  |  |
|  |  | - decentralization |  |  |  |  |  |
